# Research on the Changes to the Lipid/Polymer Membrane Used in the Acidic Bitterness Sensor Caused by Preconditioning

**DOI:** 10.3390/s16020230

**Published:** 2016-02-15

**Authors:** Yuhei Harada, Junpei Noda, Rui Yatabe, Hidekazu Ikezaki, Kiyoshi Toko

**Affiliations:** 1 Graduate School of Information Science and Electrical Engineering, Kyushu University, Nishi-ku, Fukuoka 819-0395, Japan; y.harada@nbelab.ed.kyushu-u.ac.jp (Y.H.); j.noda@nbelab.ed.kyushu-u.ac.jp (J.N.); toko@ed.kyushu-u.ac.jp (K.T.); 2 Research and Development Center for Taste and Odor Sensing, Kyushu University, Nishi-ku, Fukuoka 819-0395, Japan; 3 Intelligent Sensor Technology, Inc., 5-1-1 Onna, Atsugi-shi, Kanagawa 243-0032, Japan; Ikezaki.Hidekazu@insent.co.jp

**Keywords:** taste sensor, bitterness sensor, lipid/polymer membrane, CPA value

## Abstract

A taste sensor that uses lipid/polymer membranes can evaluate aftertastes felt by humans using Change in membrane Potential caused by Adsorption (CPA) measurements. The sensor membrane for evaluating bitterness, which is caused by acidic bitter substances such as *iso*-alpha acid contained in beer, needs an immersion process in monosodium glutamate (MSG) solution, called “MSG preconditioning”. However, what happens to the lipid/polymer membrane during MSG preconditioning is not clear. Therefore, we carried out three experiments to investigate the changes in the lipid/polymer membrane caused by the MSG preconditioning, *i.e.*, measurements of the taste sensor, measurements of the amount of the bitterness substance adsorbed onto the membrane and measurements of the contact angle of the membrane surface. The CPA values increased as the preconditioning process progressed, and became stable after 3 d of preconditioning. The response potentials to the reference solution showed the same tendency of the CPA value change during the preconditioning period. The contact angle of the lipid/polymer membrane surface decreased after 7 d of MSG preconditioning; in short, the surface of the lipid/polymer membrane became hydrophilic during MSG preconditioning. The amount of adsorbed *iso*-alpha acid was increased until 5 d preconditioning, and then it decreased. In this study, we revealed that the CPA values increased with the progress of MSG preconditioning in spite of the decrease of the amount of *iso*-alpha acid adsorbed onto the lipid/polymer membrane, and it was indicated that the CPA values increase because the sensor sensitivity was improved by the MSG preconditioning.

## 1. Introduction

Human beings perceive saltiness, sourness, bitterness, umami and sweetness when they taste foods and beverages; these are called “five basic tastes”. Human beings perceive these basic tastes with the taste buds on the tongue. Each taste bud consists of approximately 100 taste cells [1]. Sensory tests are the main method of evaluating taste even now. In sensory tests, sensory panelists really taste samples to evaluate them, however, this has some problems. One of them is low objectivity, and another is reproducibility. In addition, the tests might inflict stress on panelists. Against this background, objective evaluations of taste using sensors have attracted considerable attention [2,3,4,5,6,7].

A taste sensor that is a type of electronic tongue has been developed to evaluate the taste of foods and beverages. The taste sensor comprises potentiometric sensor electrodes with lipid/polymer membranes, each of which respond selectively to each basic taste [4,5,6,7,8,9,10,11,12]. The lipid/polymer membrane is comprised of lipids, plasticizers and a polymer. Lipids and plasticizers play the role of controlling of electric charges on the membrane surface, and the polymer is used to form the membrane. The change in the membrane potential, which is caused by the interaction between the lipid/polymer membrane and tastants, is used as the sensor output. The interactions include both the electrical interaction and the hydrophobic interaction. The variety and the amount of lipids and plasticizers are changed and adjusted to respond selectively to each taste quality. We used the change in membrane potential caused by adsorption (CPA) value as an index of the aftertastes: bitterness, astringency and umami [4,5,6,7,8,9,10,11,12].

The taste sensor as well as electronic tongues (e-tongues) have the characteristic of using in common semi-selective sensor electrodes and measuring liquid samples. The taste sensor has been developed according to the following concepts: (I) the response of the taste sensor should be similar to that of human taste; this fact is called global selectivity; (II) the taste sensor threshold for each taste should accord with the human sense; (III) the unit of taste information from the taste sensor should be clearly defined; (IV) the taste sensor can detect interactions between taste substances or taste qualities such as a suppression effect that appears between sweetness and bitterness. The taste sensor can distinguish and quantify samples into the five basic taste qualities [4,5,6,7,8,9,10,11,12]. Then again, e-tongues are designed to distinguish and analyze foods and beverages by using several types of electrodes. These electrodes have different response characteristics, and the outputs are treated with statistical analysis, e.g., principal component analysis (PCA), partial least squares (PLS) regression and neural network techniques [2,13,14,15,16,17,18,19,20,21,22]. There are many review papers about e-tongues [2,13,16]. Some of these e-tongues are based on voltammetric measurements using metallic electrodes [14,19,20] which is a very different technique from that used in taste sensors.

Among the five basic taste qualities, bitterness indicates a poison signal, and is an undesirable taste for human beings; therefore, high precision bitterness evaluation is desirable in the food and pharmaceutical industries. The taste sensor evaluates bitterness using three kinds of bitterness sensors: C00 (for acidic bitterness), BT0 (for bitterness caused by hydrochloride salts) and AN0 (for basic bitterness). C00 can be used for terpenoids such as *iso*-alpha acid. BT0 can be used for alkaloids such as quinine hydrochloride. AN0 can be used for basic drugs such as famotidine. Usually, the sensor electrodes need a preconditioning process using a reference solution comprised of 30 mmol/L KCl and 0.3 mmol/L tartaric acid for one day. This preconditioning process involves immersing the membrane in the solution for a certain period of time. In the case of the bitterness sensor C00, the sensor response, *i.e.*, the CPA value, becomes large and stable by using a preconditioning process with monosodium glutamate (MSG) solution for several days [23]. The study of the property changes of the lipid/polymer membrane used in the bitterness sensor C00 caused by MSG preconditioning is one of the approaches to clarify the mechanism of the taste sensor, and will also be instrumental for the high functionality of the taste sensor. In a previous study [24], the influence of MSG preconditioning upon the surface chemical structure of the C00 membrane was revealed. However, the reason why the CPA value increased after the MSG preconditioning process was not explained. Thus, the study of the relationship between the CPA value and the amount of taste substances adsorbed onto the C00 membrane is important for a better understanding of the C00 sensor. In this research, we investigated the changes in the C00 sensor membrane caused by MSG preconditioning by performing three kinds of experiments which included the measurement of the amount of adsorption.

## 2. Experimental Section

### 2.1. Reagents

Tetradodecylammonium bromide (TDAB) was obtained from Sigma-Aldrich, Inc. (St. Louis, MO, USA). 2-Nitrophenyloctyl ether (NPOE) was purchased from Dojindo Laboratories (Kumamoto, Japan). Polyvinyl chloride (PVC) was obtained from Wako Pure Chemical Industries, Ltd. (Osaka, Japan). *Iso*-alpha acid was obtained from Intelligent Sensor Technology Inc. (Kanagawa, Japan). Monosodium glutamate (MSG), Potassium chloride (KCl) and tartaric acid were obtained from Kanto Chemical Co., Inc. (Tokyo, Japan). All aqueous solutions for experiments were prepared with deionized water.

### 2.2. Lipid/Polymer Membrane

**Figure 1:**
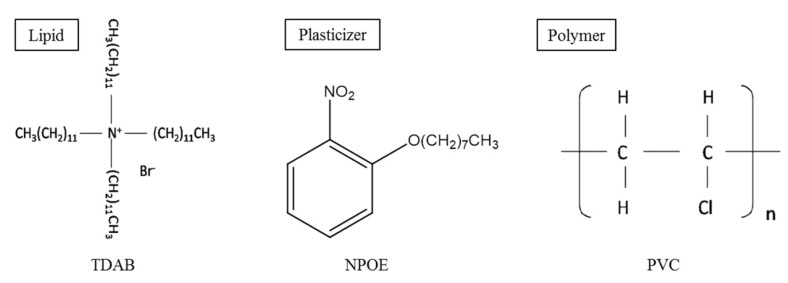
The chemical structures of TDAB, NPOE and PVC.

A lipid/polymer membrane is used in a commercialized taste sensor, the TS-5000Z, for evaluating acidic bitterness C00. The lipid/polymer membrane is composed of TDAB as the lipid, NPOE as the plasticizer and PVC as the supporting polymer (Figure 1). This membrane comprises below 1 wt% of the lipid, as shown by the previous results [11], and it is supposed to be hydrophobic.

In [8], we reported that this sensor showed high selectivity to an acidic bitter substance, *iso*-alpha acid, by using the CPA measurement. The CPA values corresponding to saltiness, sourness, umami, basic bitterness, astringency and sweetness were nearly zero. We fabricated the membranes using the following procedure used in the previous studies [4,5,6,7,8].

### 2.3. Measurement Procedure of Taste Sensor

**Figure 2:**
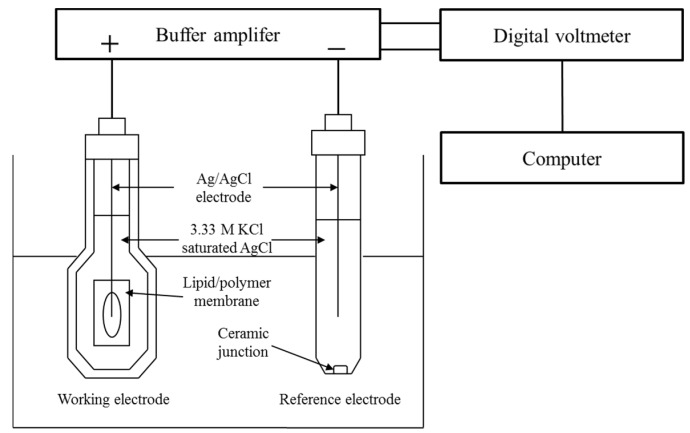
The measurement system of the taste sensor.

We used the TS-5000Z taste sensing system (Intelligent Sensor Technology, Inc., Kanagawa, Japan) for this measurement (Figure 2). Several sensor electrodes and a reference electrode (Ag/AgCl electrode) are attached to the taste sensor. It measures the changes in the membrane potential that are generated when the sensor electrodes are soaked in a sample solution. We used an *iso*-alpha acid solution as the acidic bitterness sample solution, and the solvent is a reference solution comprised of 30 mmol/L KCl and 0.3 mmol/L tartaric acid. *Iso*-alpha acid is the extract from hops, and the main components are presumed to be *cis-* and *trans*-isohumulone. The concentration of the *iso*-alpha acid solution in all experiments is 0.01 vol%. The measurement procedure [7,8,9,10,11,12] is as follows: the first step is soaking the sensor electrode in the reference solution to measure the membrane potential for the reference solution, Vr. The next step is soaking the sensor electrode in the sample solution to measure the membrane potential for the sample solution, Vs. Then the difference, Vs-Vr, is defined as the relative value. After that, the sensor electrode is soaked once more in another reference solution to measure the membrane potential for the reference solution, Vr'. Then the difference, Vr'-Vr, is defined as the CPA value. Finally, the membrane is rinsed with a rinsing solution consisting of 100 mmol/L KCl, 10 mmol/L KOH, 30 vol% ethanol. We repeated this procedure five times for each sample.

### 2.4. Measurement of Contact Angle

We measured the contact angle of the surface of the lipid/polymer membrane by a contact angle meter (DM500, Kyowa Interface Science Co., Ltd., Saitama, Japan). The lipid/polymer membrane was cut and set on a slide glass on the contact angle meter. The contact angle was measured with a 2 μL water droplet. We repeated the measurement three times to obtain each datapoint.

### 2.5. Measurement of Amount of Adsorbed Iso-Alpha Acid

**Figure 3:**
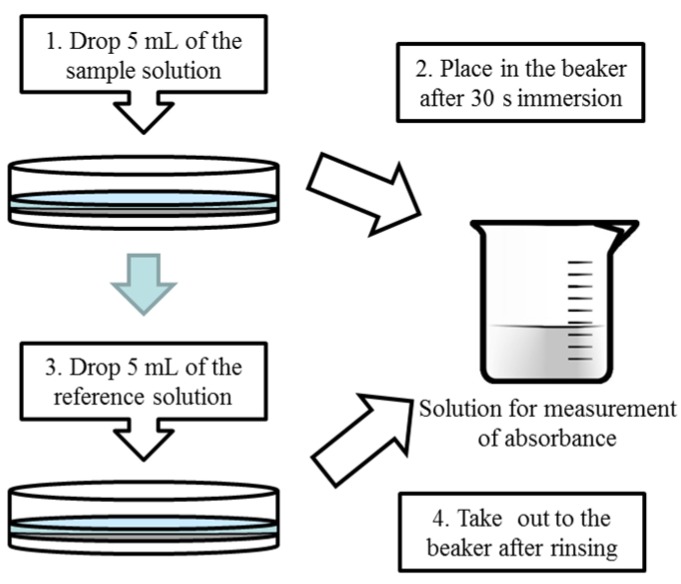
Measurement procedure of the amount of adsorbed onto the membrane.

We used an UV-visible spectrometer (UV-1800, Shimadzu Corp., Kyoto, Japan) for measurement of the amount of *iso*-alpha acid adsorbed onto the lipid/polymer membrane. This measurement is an indirect method to evaluate the amount of adsorption. We measured the amount of taste substances that were not adsorbed onto the lipid/polymer membrane in the sample solution because of the difficulty of measuring directly the amount of taste substances adsorbed onto the lipid/polymer membrane. The measurement procedure was slightly changed from previous studies [9,10,11,12] in order to increase the accuracy, shown as in Figure 3. There are five steps in this measurement: first, a calibration curve was obtained using an *iso*-alpha solution of known concentration. Next, 5 mL of the *iso*-alpha acid solution was added onto the lipid/polymer membrane formed in a Petri dish, and kept for 30 s, which would lead to adsorption of the *iso*-alpha acid onto the lipid/polymer membrane during this period. After that, the *iso*-alpha acid solution was placed in a beaker. Next, the lipid/polymer membrane was rinsed with 5 mL of the reference solution, which was added to the same beaker. Finally, the absorbance of the solution in the beaker was measured and the concentration of this solution was calculated by a calibration curve. We calculated the amount of adsorbed *iso*-alpha acid from the difference between the concentration of *iso*-alpha acid before and after dropping. This value was divided by the size of the Petri dish to obtain the amount of *iso*-alpha acid adsorbed per square centimeter.

## 3. Results and Discussion

### 3.1. Response of Taste Sensor

As mentioned above, the taste sensor for acidic bitterness needs an immersion process in MSG solution (30 mmol/L in solution) for several days. Previous studies [8,24] showed that the sensor response to 0.01 vol% *iso*-alpha acid increases as the MSG preconditioning progresses, and becomes about −80 mV in the stable region after several days of MSG preconditioning. In this study, we carried out 7 d of preconditioning and five measurements each day.

**Figure 4:**
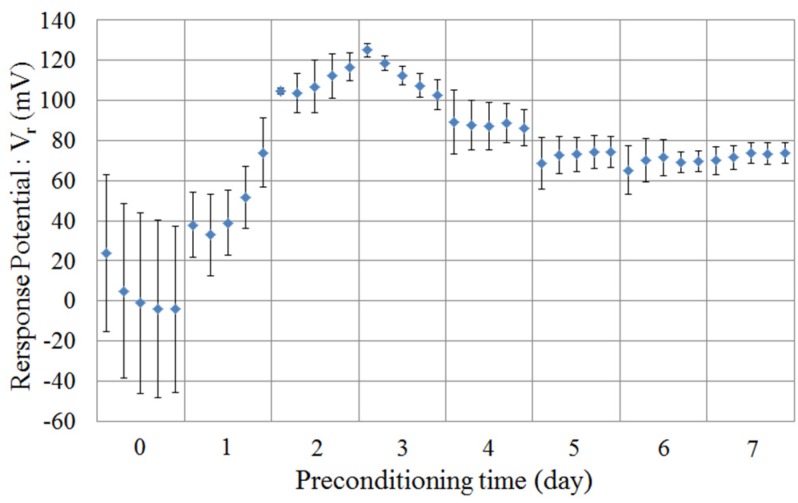
Relationship between the response potential to the reference solution (30 mmol/L KCl + 0.3 mmol/L tartaric acid) and the preconditioning time. Data are expressed as mean ± SD (n = 4).

Figure 4 shows the change of the response potential to the reference solution (Vr) caused by preconditioning. The magnitude of the response potential was small, but the errors were large for the first two days. After 2 or 3 d, the response potential increased and became stable. This tendency of the change of the response potential is similar to that of the change of the CPA values. This result shows the same tendency the previous study [24].

**Figure 5:**
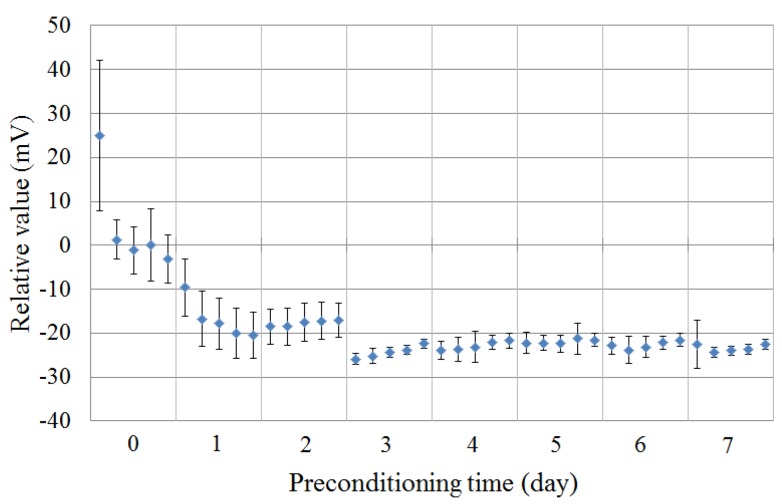
Relationship between the relative value to the saltiness sample (300 mmol/L KCl + 0.3 mmol/L tartaric acid) and the preconditioning time. Data are expressed as mean ± SD (n = 4).

Figure 5 shows the change of the relative value to the saltiness sample (300 mmol/L KCl + 0.3 mmol/L tartaric acid) caused by preconditioning. The relative values were below −20 mV at first, and the error bars were large. The magnitude of the relative value increased and became stable after 2 or 3 d of preconditioning. This tendency of the change of the relative value is similar to that of the change of the response potential to the reference solution.

There are many reports concerned with the electrochemical mechanism of the membrane potential [25,26,27,28,29]. In this study, we focused on the change of the membrane potential caused by MSG preconditioning, and discuss it. The transition region in Figure 4 is supposed to occur as follows: in [24], in which a lipid/polymer membrane of the same composition was used, it was revealed that TDAB is concentrated on the lipid/polymer membrane surface and that MSG is adsorbed onto the lipid/polymer membrane surface after 7 d of MSG preconditioning. This concentration of TDAB on the membrane surface is a factor causing the surface of the lipid/polymer membrane to be positively charged because TDAB is a positively charged lipid. In contrast, the adsorption of MSG on the membrane surface is a factor causing the decrease of the surface charge density of the lipid/polymer membrane because MSG, which is negatively charged in the MSG solution, neutralizes the positive charge on the membrane surface. Initially, the concentration of TDAB mainly causes the surface charge density to change. Therefore, the response potential to the reference solution increased at first. As the surface charge density of the membrane is increased, MSG is more attracted to the membrane surface and the adsorption of MSG is increased. This causes the decrease of the response potentials to the reference solution. After that, the concentrating TDAB is balanced with the adsorption of MSG, and the response potentials to the reference solution become stable. In summary, the electrical characteristics of the C00 sensor membrane were improved by the MSG preconditioning.

### 3.2. Contact Angle of Membrane Surface

**Figure 6:**
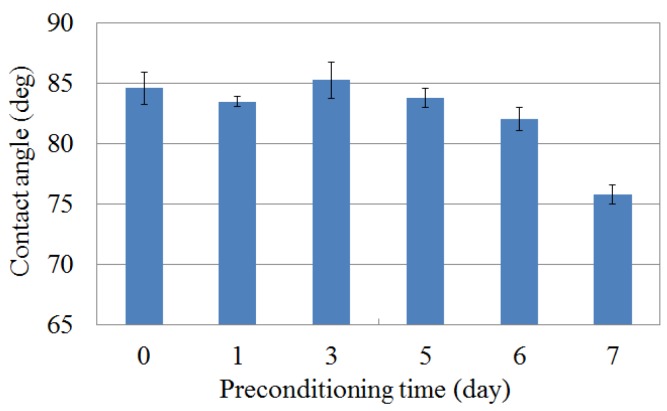
Relationship between the contact angle of the membrane surface and the preconditioning time. Data are expressed as mean ± SD (n = 3).

The contact angle is used as an index of the hydrophobicity in the solid surface. A large contact angle indicates a strong hydrophobicity, and a small contact angle indicates a weak hydrophobicity. Figure 6 shows the contact angle of the lipid/polymer membrane surface. The change of the contact angle was small until 5 d of preconditioning, and the contact angle decreased at 7 d of preconditioning. This result indicates that the hydrophobicity of the lipid/polymer membrane surface were maintained until 5 d of MSG preconditioning, and became weakened by MSG preconditioning. The results corresponding to 6 and 7 d of MSG preconditioning show the same tendency as the previous study [24]. The decrease of the contact angle can be considered to be caused by the adsorption of the MSG molecules onto the membrane surface [24]. In summary, the lipid/polymer membrane surface was hydrophobic at first, and became hydrophilic after MSG preconditioning.

**Figure 7:**
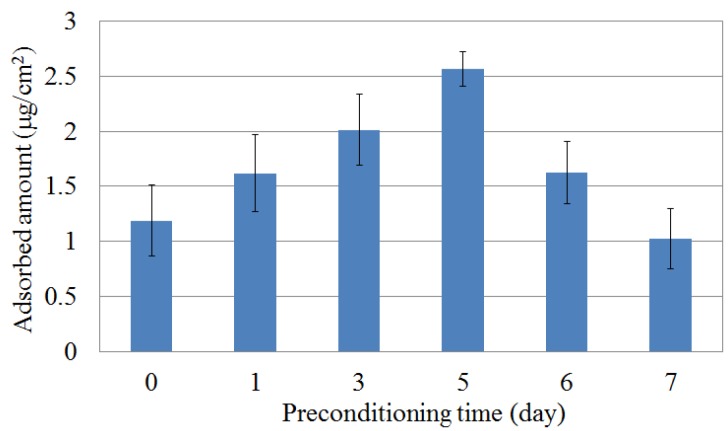
Relationship between the amount of adsorbed *iso*-alpha acid onto lipid/polymer membrane and the preconditioning time. Data are expressed as mean ± SD (n = 6).

*Iso*-alpha acid tends to be adsorbed onto the hydrophobic surface by the hydrophobic interaction after it is attracted by the electrical interaction. At first, the membrane surface is nearly neutral or is slightly positively charged. Therefore, the electrical interaction between the membrane surface and *iso*-alpha acid is weak, and the amount of *iso*-alpha acid adsorbed onto the lipid/polymer membrane is small. As the surface charge density is increased during the preconditioning progress, as understood from Figure 4 and Figure 5, the electrical interaction between the membrane surface and *iso*-alpha acid become strong and the amount of *iso*-alpha acid adsorbed onto the lipid/polymer membrane increases. After that, the membrane surface becomes hydrophilic, caused by the adsorption of MSG, as understood from Figure 7, and the amount of *iso*-alpha acid adsorbed onto the lipid/polymer membrane decreases. Therefore, these results indicate that the adsorbed amount onto lipid/polymer membrane is decided by the surface charge density and the hydrophobicity of the lipid/polymer membrane.

In previous studies [9,10,11,12], there existed a relationship between the CPA value and the amount of taste substances adsorbed onto the lipid/polymer membrane. In the present experiment, however, the CPA values remained high after 5 d preconditioning, whereas the amount of adsorbed *iso*-alpha acid decreased. The reason is supposed to be as follows: as mentioned above, the amount of adsorbed *iso*-alpha acid was decreased by the adsorption of MSG. At the same time, the adsorption of MSG caused a decrease of the surface charge density of the lipid/polymer membrane, as described in Section 3.1. As the surface charge density is decreased, the membrane surface becomes sensitive to the adsorption of *iso*-alpha acid. As a result, the CPA values remain at high levels regardless of the decrease of the adsorption amount. In summary, the amount of *iso*-alpha acid adsorbed onto lipid/polymer membrane is increased up to 5 d of preconditioning and is decreased after 6 d of MSG preconditioning.

## 4. Conclusions

We examined the influence of MSG preconditioning on the lipid/polymer membrane used in the acidic bitterness sensor C00. First, we measured the *iso*-alpha acid using the taste sensor. The response potentials to the reference solution increased as the preconditioning process progressed, and became stable after 4 days of preconditioning. The response potentials to the reference solution were affected by two factors: the concentrating TDAB and the adsorption of MSG. Next, we measured the contact angle of the lipid/polymer membrane surface. The results showed that the surface of the lipid/polymer membrane was hydrophobic until 5 d of MSG preconditioning, and became hydrophilic after 7 d of MSG preconditioning. Lastly, we measured the amount of *iso*-alpha acid adsorbed onto the lipid/polymer membrane. The amount of adsorbed *iso*-alpha acid increased until 5 d of preconditioning, after that, it decreased. It was suggested that the CPA values remained at high levels even though the amount of the adsorbed *iso*-alpha acid decreased because the sensitivity of the C00 membrane was improved by MSG preconditioning. In this study, we clarified that the increase of the CPA value in the bitterness sensor C00 caused by MSG preconditioning is not caused by the change of the amount of *iso*-alpha acid adsorbed onto the lipid/polymer membrane and it was indicated that the reason why the CPA values increased is the improvement of the sensor sensitivity caused by MSG preconditioning.

## Abbreviations

The following abbreviations are used in this manuscript:

CPA: change in membrane potential caused by adsorption

MSG: monosodium glutamate

TDAB: tetradodecylammonium bromide

NPOE: 2-nitrophenyloctyl ether

PVC: polyvinyl chloride

## References

[1] Chandrashekar J., Hoon M.A., Ryba N.J.P., Zuker C.S. (2006). The receptors and cells for mammalian taste. Nature.

[2] Riul A., Dantas C.A.R., Miyazaki C.M., Oliveira O.N. (2010). Recent advances in electronic tongues. Analyst.

[3] Savage N. (2012). Technology: The taste of things to come. Nature.

[4] Toko K. (2013). Biochemical Sensors: Mimicking Gustatory and Olfactory Senses.

[5] Toko K. (2000). Biomimetic Sensor Technology.

[6] Kobayashi Y., Habara M., Ikezaki H., Chen R., Naito Y., Toko K. (2010). Advanced taste sensors based on artificial lipids with global selectivity to basic taste qualities and high correlation to sensory scores. Sensors.

[7] Tahara Y., Toko K. (2013). Electronic tongues—A review. IEEE Sens. J..

[8] Habara M., Toko K. (2006). Taste sensor. Encyclopedia of Sensors.

[9] Fukagawa T., Tahara Y., Yasuura M., Habara M., Ikezaki H., Toko K. (2012). Relationship between taste sensor response and amount of quinine adsorbed on lipid/polymer membrane. J. Innov. Electron. Commun..

[10] Hara D., Fukagawa T., Tahara Y., Yasuura M., Toko K. (2014). Examination of amount of astringent substances adsorbed onto lipid/polymer membrane used in taste sensor. Sens. Lett..

[11] Toko K., Hara D., Tahara Y., Yasuura M., Ikezaki H. (2014). Relationship between the amount of bitter substances adsorbed onto lipid/polymer membrane and the electric response of taste sensors. Sensors.

[12] Harada Y., Tahara Y., Toko K. (2015). Study of the relationship between taste sensor response and the amount of epigallocatechin gallate adsorbed onto a lipid-polymer membrane. Sensors.

[13] Anand V., Kataria M., Kukkar V., Saharan V., Choudhury P.K. (2007). The latest trends in the taste assessment of pharmaceuticals. Drug Discov. Today.

[14] Winquist F. (2008). Voltammetric electronic tongues—Basic principles and applications. Microchim. Acta.

[15] Vlasov Y., Legin A., Rudnitskaya A., di Natale C., D’Amico A. (2005). Nonspecific sensor arrays (“electronic tongue”) for chemical analysis of liquids. Pure Appl. Chem..

[16] Ciosek P., Wroblewski W. (2007). Sensor arrays for liquid sensing-electronic tongue systems. Analyst.

[17] Legin A., Rudnitskaya A., Vlasov Y., di Natale C., Davide F., D’Amico A. (1997). Tasting of beverages using an electronic tongue. Sens. Actuators B Chem..

[18] Citterio D., Suzuki K. (2008). Smart taste sensors. Anal. Chem..

[19] Ivarsson P., Krantz-Rülcker C., Winquist F., Lundström I. (2005). A voltammetric electronic tongue. Chem. Sens..

[20] Winquist F., Wide P., Lundström I. (1997). An electronic tongue based on voltammetry. Anal. Chim. Acta.

[21] Vlasov Y., Bychkov E.A., Legin A. (1994). Chalcogenide glass chemical sensors: Research and analytical applications. Talanta.

[22] Vlasov Y., Legin A., Rudnitskaya A. (1997). Cross-sensitivity evaluation of chemical sensors for electronic tongue: Determination of heavy metal ions. Sens. Actuators B Chem..

[23] Insent, Inc. (2007). Highly Durable and Rapidly Measurable Taste Sensor for Quality Control of Products Using Artificial Lipid/Polymer Membrane.

[24] Yatabe R., Noda J., Tahara Y., Naito Y., Ikezaki H., Toko K. (2015). Analysis of a lipid/polymer membrane for bitterness sensing with a preconditioning process. Sensors.

[25] Kamo N., Oikawa M., Kobatake Y. (1973). Effective fixed charge density governing membrane phenomena V. A reduced expression of permselectivity. J. Phys. Chem..

[26] Oohira K., Toko K. (1996). Theory of electric characteristics of the lipid/PVC/DOPP membrane and PVC/DOPP membrane in response to taste stimuli. Biophys. Chem..

[27] Kamo N., Kobatake Y. (1974). Interpretation of asymmetric membrane potential. J. Colloid Interface Sci..

[28] Eric B., Philippe B., Ernö P. (2004). The phase-boundary potential model. Talanta.

[29] Johan B., Ari I., Andrzej L. (2008). Potentiometric Ion Sensors. Chem. Rev..

